# Should Remaining Stockpiles of Smallpox Virus (Variola) Be Destroyed?

**DOI:** 10.3201/eid1704.101865

**Published:** 2011-04

**Authors:** Raymond S. Weinstein

**Affiliations:** Author affiliation: Georgetown University School of Medicine, Washington, DC, USA; George Mason University, Manassas, Virginia, USA

**Keywords:** Smallpox, variola, vaccinia, poxvirus, immunology, WHO, policy review

## Abstract

In 2011, the World Health Organization will recommend the fate of existing smallpox stockpiles, but circumstances have changed since the complete destruction of these cultures was first proposed. Recent studies suggest that variola and its experimental surrogate, vaccinia, have a remarkable ability to modify the human immune response through complex mechanisms that scientists are only just beginning to unravel. Further study that might require intact virus is essential. Moreover, modern science now has the capability to recreate smallpox or a smallpox-like organism in the laboratory in addition to the risk of nature re-creating it as it did once before. These factors strongly suggest that relegating smallpox to the autoclave of extinction would be ill advised.

In 2011, the World Health Organization (WHO) plans to announce its recommendation regarding the final destruction of all known remaining smallpox virus stockpiles. Smallpox, an ancient human scourge of unparalleled destructive importance throughout most of recorded human history (Figure 1), is believed to have emerged in the Middle East some 6,000–10,000 years ago (*1**,**2*) from either camelpox or the gerbil-specific taterapox (*3**–**5*). It holds a status as one of the great killers in all human history, having produced the horrific deaths of up to 500 million persons in just the 20th century alone (*6*). At first glance, the answer to this conundrum—whether or not smallpox should be forever relegated to the autoclave of extinction—might seem an easy one. Beaten back by the Jenner vaccine first proposed in 1796, smallpox was finally declared eradicated in 1980, in one of the most profound public health achievements in human history. Since that time, WHO has made it generally known that they would like to see the elimination of all remaining variola stockpiles and made the United States and Russia the repository for all remaining stocks. At the 60th Annual World Health Assembly in 2007, the organization postponed the final decision for any recommended destruction deadline until their next meeting in 2011.

**Figure 1:**
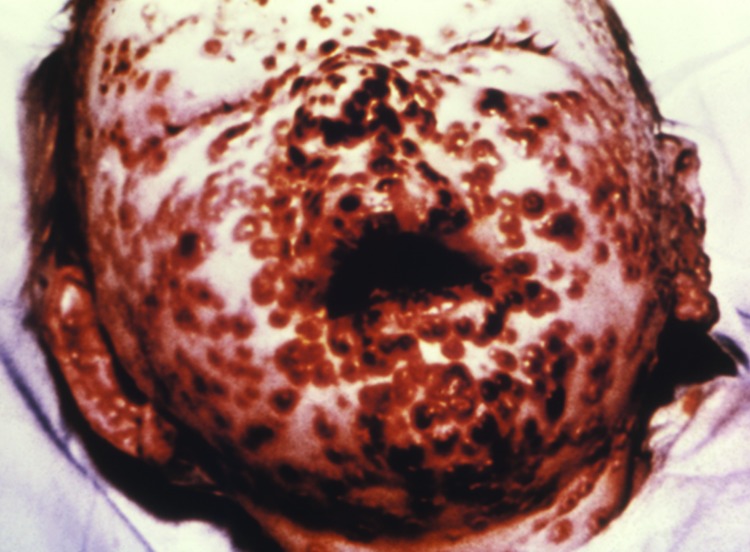
One-year-old child on day 10 of a smallpox infection; his face is covered with painful lesions that are beginning to scab. Photograph courtesy of the Centers for Disease and Prevention Public Health Image Library; by Charles Farmer, Jr., 1962.

The last officially acknowledged stocks of variola are held by the United States at the Centers for Disease Control and Prevention and by Russia at the State Research Centre of Virology and Biotechnology. The US collection consists of 450 isolates of variola, while various authoritative sources place the number of specimens retained by Russia at ≈150 samples, consisting of 120 different strains (*7**,**8*), including several selected for their increased virulence that were collected during the Cold War as potential biological weapons. The possibility that stolen smallpox cultures may already be in the hands of rogue states or terrorist organizations also remains an important subject of international concern.

Even though in 1980, then Secretary of Health and Human Services Louis Sullivan promised the destruction of US variola stockpiles within 3 years (*8*), this has not yet occurred in either the United States or Russia, and no actual recommendation for destruction has been issued by the World Health Assembly. To understand the reasons behind this apparent hesitance to once and for all eliminate from existence all remaining traces of the smallpox virus, one has to understand how the implications of this action have changed over the past several decades in a scientific world decidedly different from the one in which the idea of smallpox virus destruction was first proposed.

Currently, the only real benefit to destroying all known remaining stockpiles of variola in the world would be the elimination of the extremely unlikely possibility of unleashing a lethal epidemic due to the theft or accidental release of the virus from one of the remaining official stocks. In reality, this destruction would provide only an illusion of safety, and the drawbacks are many.

The prolonged existence of smallpox, combined with the important clinical implications of its high infectivity and mortality rates, suggests that the human immune system evolved under the disease’s considerable evolutionary influence. In the past decade, for example, advances in immunologic research have suggested that the variola virus and its close relative and experimental surrogate, vaccinia, have a remarkable ability to substantially alter the immune response of its human host (*9*). Genomic and proteomic analysis and microarray surveys have demonstrated immunologic targets of smallpox that include, at minimum, several chemokines and their receptors, interleukin-8, interferon-γ, tumor necrosis factor–α, and the downstream target of receptor NFκB, and multiple components of the complement cascade (*10**–**15*). Although we are only just beginning to unravel the complex pathophysiology and virulence mechanisms of smallpox virus, experimental evidence with vaccinia has also demonstrated that many of the observed immunologic alterations produced by poxvirus infection persist long term and can be measured months or years after infection (*9*).

In addition, the evolutionary success of the CC-chemokine receptor null mutation, CCR5^Δ32^, believed to have first appeared in northern Europe up to 3,500 years ago in a single person, is a good example of the importance of smallpox in human immune evolution (*16*). Today the mutation can be found in ≈10% of all those of northern European descent, preventing expression of the as-yet mostly inscrutable CCR5 receptor on the surface of many different subsets of immune cells. The huge success of this mutation is most likely because of the survival advantage it conferred by providing a marked resistance to smallpox (*16**–**18*). Notably, this same mutation confers nearly complete immunity to HIV. A recently published study suggests that the apparently sudden emergence and explosive spread of HIV may be related to the eradication of smallpox, postulating that widespread exposure to both variola and vaccinia (the virus that comprises the smallpox vaccine [Figure 2]) may have previously inhibited the successful spread of HIV (*19*). The immunologic mechanisms underlying this intriguing, and potentially useful, effect remain elusive. Thus, eliminating all known remaining smallpox stocks might hinder ongoing research in this direction.

**Figure 2:**
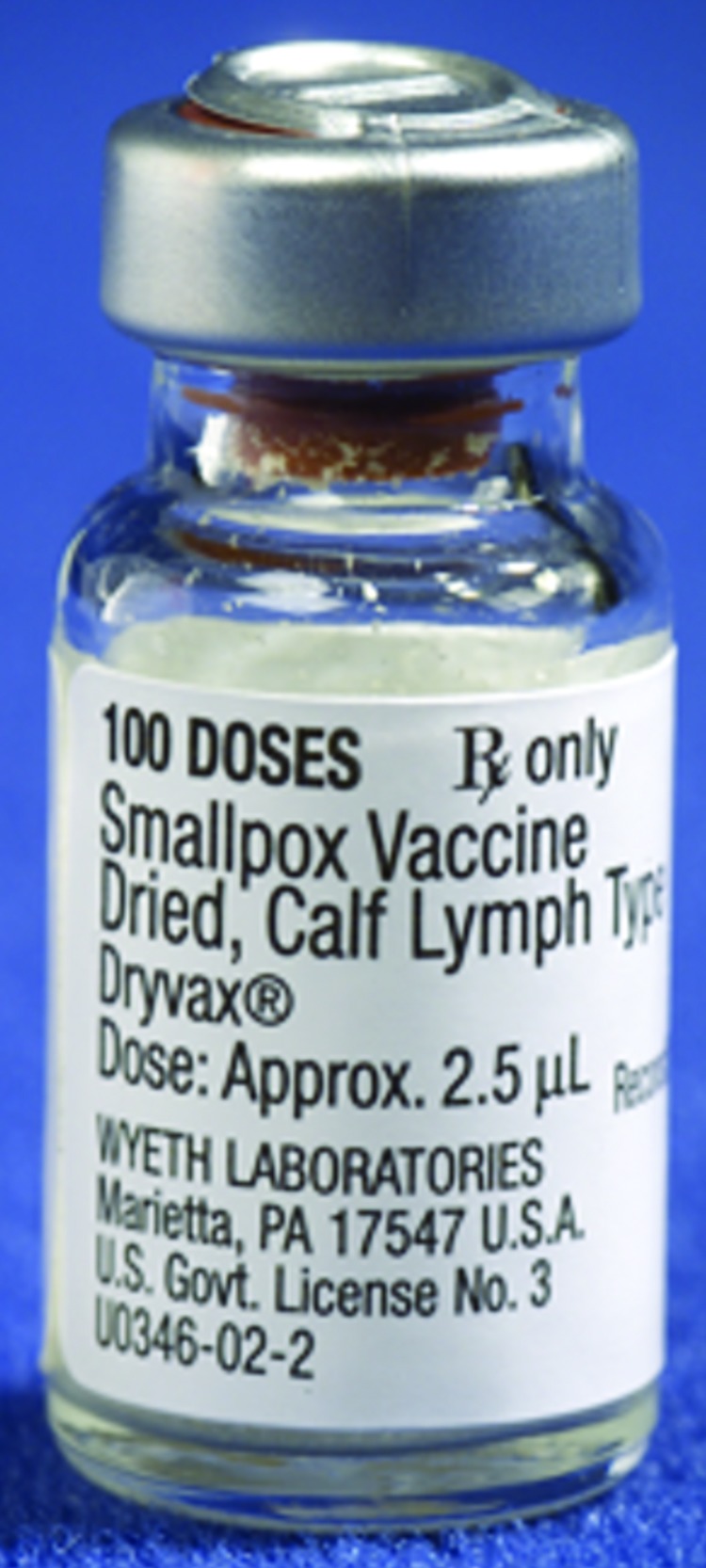
The no-longer-manufactured Wyeth vaccine that made possible the ultimate eradication of smallpox. Photograph courtesy of the Centers for Disease Control and Prevention Public Health Image Library; by James Gathany, 2002.

The immune alterations produced by smallpox can serve as a window and guide to previously unappreciated immunologic mechanisms, the full understanding of which might lead to new therapeutic options for a host of diseases, both infectious and autoimmune. No one can yet be certain what role, if any, an intact variola virus might play in future research, and in providing important new insights into the human immune response as well as into the malevolence of this virus and related viruses. It is certain, however, that if the last remaining stockpiles are destroyed, the door to any possibility of future research employing the virus will be forever and irreversibly shut.

Finally, today’s science is capable, through genetic manipulation, of re-creating a highly virulent smallpox-like virus from a closely related poxvirus or even from scratch. But perhaps what we should fear even more is nature creating it for us, as it so efficiently did once before from the still-existent progenitors of variola. The possibility is certainly not unthinkable that nature could once again fashion smallpox from a near relative poxvirus or even create a new, smallpox-like human pathogen from a clinically similar but more genetically divergent zoonotic poxvirus, such as monkeypox. Several recent reviews have reported an increasing prevalence of human monkeypox since smallpox eradication and the cessation of vaccinia vaccination (*20**,**21*). The possible re-creation of smallpox by either natural or modern laboratory means would render moot any argument regarding the destruction of remaining stockpiles of smallpox virus in the mistaken belief that it would be for the benefit and protection of mankind.

## References

[1] Barquet N, Domingo P. Smallpox: the triumph over the most terrible of the ministers of death. Ann Intern Med. 1997;127:635–42. 9341063 10.7326/0003-4819-127-8_part_1-199710150-00010

[2] Shchelkunov SN. How long ago did smallpox virus emerge? Arch Virol. 2009;154:1865–71. 10.1007/s00705-009-0536-0 19882103

[3] Afonso CL, Tulman ER, Lu Z, Zsak L, Sandybaev NT, Kerembekova UZ, The genome of camelpox virus. Virology. 2002;295:1–9. 10.1006/viro.2001.1343 12033760

[4] Li Y, Carroll DS, Gardner SN, Walsh MC, Vitalis EA, Damon IK. On the origin of smallpox: correlating variola phylogenics with historical smallpox records. Proc Natl Acad Sci U S A. 2007;104:15787–92. 10.1073/pnas.0609268104 17901212 PMC2000395

[5] Afonso CL, Tulman ER, Lu Z, Zsak L, Sandybaev NT, Kerembekova UZ, The genome of camelpox virus. Virology. 2002;295:1–9. 10.1006/viro.2001.1343 12033760

[6] Koplow DA. Smallpox: the fight to eradicate a global scourge. Berkeley (CA): University of California Press; 2003.

[7] World Health Organization Advisory Committee on Variola Virus Research. WHO report of the ninth meeting, Geneva, Switzerland, November 29–30, 2007. Geneva: The Organization; 2008. p. 2.

[8] Mahy BWJ, Almond JW, Berns KI, Chanock RM, Lvov DK, Pettersson RF, The remaining stocks of smallpox virus should be destroyed. Science. 1993;262:1223–4. 10.1126/science.8235651 8235651

[9] Brichacek B, Vanpouille C, Trachtenberg AJ, Pushkarsky T, Dubrovsky L, Martin G, Long-term changes of serum chemokine levels in vaccinated military personnel. BMC Immunol. 2006;7:21. 10.1186/1471-2172-7-21 16965634 PMC1578581

[10] Rubins KH, Hensley LE, Jahrling PB, Whitney AR, Geisbert TW, Huggins JW, The host response to smallpox: analysis of the gene expression program in peripheral blood cells in a nonhuman primate model. Proc Natl Acad Sci U S A. 2004;101:15190–5. 10.1073/pnas.0405759101 15477590 PMC523453

[11] Mohamed MR, Rahman MM, Lanchbury JS, Shattuck D, Neff C, Proteomic screening of variola virus reveals a unique NFκB inhibitor that is highly conserved among pathogenic orthopoxviruses. Proc Natl Acad Sci U S A. 2009;106:9045–50. 10.1073/pnas.0900452106 19451633 PMC2683884

[12] Alejo A, Ruiz-Arguello MB, Ho Y, Smith VP, Saraiva M, Alcami A, A chemokine-binding domain in the tumor necrosis factor receptor from variola (smallpox) virus. Proc Natl Acad Sci U S A. 2006;103:5995–6000. 10.1073/pnas.0510462103 16581912 PMC1458686

[13] Dunlop LR, Oehlberg KA, Reid JJ, Avci D, Rosengard AM. Variola virus immune evasion proteins. 2003. Microbes Infect. 2003;5:1049–56. 10.1016/S1286-4579(03)00194-1 12941397

[14] McFadden G. Smallpox: an ancient disease enters the modern era of virogenomics. Proc Natl Acad Sci U S A. 2004;101:14994–5. 10.1073/pnas.0406207101 15479762 PMC524071

[15] Antonets DV, Nepomnyashchikh TS, Shchelkunov SN. SECRET domain of variola virus CrmB protein can be a member of poxviral type II chemokine-binding proteins family. BMC Research Notes. 2010;3:271. 10.1186/1756-0500-3-271 20979600 PMC2987869

[16] Hummel S, Schmidt D, Herrmann B, Oppermann M. Detection of the CCR5-D32 HIV resistance gene in Bronze Age skeletons. Genes Immun. 2005;6:371–4. 10.1038/sj.gene.6364172 15815693

[17] Rahbar R, Murooka TT, Hinek AA, Galligan CL, Sassano A, Yu C, Vaccinia virus activation of CCR5 invokes tyrosine phosphorylation signaling events that support virus replication. J Virol. 2006;80:7245–59. 10.1128/JVI.00463-06 16809330 PMC1489052

[18] Galvani AP, Slatkin M. Evaluating plague and smallpox as historical selective pressures for the CCR5-Δ32 HIV-resistance allele. Proc Natl Acad Sci U S A. 2003;100:15276–9. 10.1073/pnas.2435085100 14645720 PMC299980

[19] Weinstein RS, Weinstein MM, Alibek K, Bukrinsky M, Brichacek B. Significantly reduced CCR5-tropic HIV-1 replication in vitro in cells from subjects previously immunized with vaccinia virus. BMC Immunol. 2010;11:23. 10.1186/1471-2172-11-23 20482754 PMC2881106

[20] Karem KL, Reynolds M, Hughes C, Braden Z, Nigam P, Crotty S, Monkeypox-induced immunity and failure of childhood smallpox vaccination to provide complete protection. Clin Vaccine Immunol. 2007;14:1318–27. 10.1128/CVI.00148-07 17715329 PMC2168110

[21] Rimoin AW, Mulembakani PM, Johnston SC, Lloyd-Smith JO, Kisalu NK, Kinkela TL, Major increase in human monkeypox incidence 30 years after smallpox vaccination campaigns cease in the Democratic Republic of Congo. Proc Natl Acad Sci U S A. 2010;107:16262–7. 10.1073/pnas.1005769107 20805472 PMC2941342

